# The Effects of Sustained Literacy Engagement on Cognition and Sentence Processing Among Older Adults

**DOI:** 10.3389/fpsyg.2022.923795

**Published:** 2022-07-11

**Authors:** Elizabeth A. L. Stine-Morrow, Giavanna S. McCall, Ilber Manavbasi, Shukhan Ng, Daniel A. Llano, Aron K. Barbey

**Affiliations:** ^1^Beckman Institute, University of Illinois, Urbana, IL, United States; ^2^Department of Educational Psychology, University of Illinois, Champaign, IL, United States; ^3^Department of Psychology, University of Illinois, Champaign, IL, United States

**Keywords:** cognitive aging, literacy, engagement, reading, plasticity

## Abstract

Considerable evidence suggests that language processing depends on memory processes, which are vulnerable to declines with aging. Yet little is known about the effects of language processing in the form of sustained literacy engagement on memory and other aspects of cognition. In the current study, adults (60–79 years of age) were randomly assigned to an 8-week program of leisure reading (*n* = 38) or to an active puzzle control (*n* = 38). Relative to the control, the experimental group showed differential improvement in verbal working memory and episodic memory. The experimental group also showed evidence of enhanced conceptual integration in sentence processing. These effects did not vary as a function of personality characteristics (e.g., openness) hypothesized to be compatible with literacy engagement. These findings support the idea that the exercise of cognitive capacities in the context of everyday life may offset age-related impairment in areas of cognition engaged by the activity, regardless of dispositional fit.

## Introduction

In broad terms, cognition in adulthood is often characterized as dynamic change in two competing forces (Baltes, 1997; Baltes et al., 1999; Finkel et al., 2007). Fluid ability (or “mental mechanics”; g_*f*_), the ability to quickly transform information and effectively control attention to respond to changing task demands, shows a monotonic decline through adulthood, as a consequence of genetically mediated senescence processes. Crystallized ability (g_*c*_), grounded in knowledge and acculturation, increases as a consequence of experience. Developmental patterns of change in the psychometric assessment of abilities is of special interest insofar as these abilities are predictive of significant everyday outcomes (Gottfredson, 1997; Kuncel et al., 2004; Deary, 2008; Deary et al., 2010; Gross et al., 2011; Gothe et al., 2014).

Even though age-related declines in fluid abilities are normative, there is considerable inter-individual variability in trajectories of change, such that some individuals age quite well (Livingston et al., 2017, 2020). Some of this variance is certainly attributable, as with virtually any biological process, to variation inherent in senescence (Hayflick, 1998), but it is also well-accepted that cognition can be enriched through experience (Hertzog et al., 2008). At the same time, theoretically grounded, empirically supported pathways to robust cognition with aging that can be translated to application remain to be discovered (Stine-Morrow et al., 2021; Stine-Morrow and Manavbasi, 2022). In fact, cognitive interventions, focused on instruction and practice in component skills, tend to produce change that is narrowly tied to the trained skill (Simons et al., 2016). Thus, there is growing interest in how activity engagement in the ecology of everyday life can broadly shape cognitive health (Park et al., 2014; Stine-Morrow et al., 2014; Carlson et al., 2015; Banducci et al., 2017; Cerino et al., 2020). In the current paper, we report the results of an investigation into the effects of everyday reading on cognition and language-related processes, as well as on dispositional factors that may sustain activity engagement. By way of introduction, we summarize what is known about cognitive enrichment with aging, as well as about the particular effects of sustained literacy engagement.

Plasticity refers to the capacity for mind and brain to be shaped by experience. While early models of development focused on plasticity as predominantly a property of young organisms (Waddington, 1942; Gottlieb, 1991), it is now understood that, while perhaps more sluggish with aging, plasticity exists throughout the lifespan (Baltes, 1997; Lövdén et al., 2010).

The clearest literature in defining causal mechanisms of plasticity through mental exercise is that in which participants are randomly assigned to groups trained in particular cognitive skills, and change in performance is measured on the skills that are specifically trained and those that are more or less similar to the targeted skills (“near” and “far” transfer). An extensive base of research with healthy older adult populations has clearly demonstrated reliable improvements in the specific skill that is trained but limited evidence for transfer to related skills or to everyday outcomes (Blieszner et al., 1981; Schaie and Willis, 1986; Willis and Nesselroade, 1990; Ball et al., 2002; Willis et al., 2006; Rebok et al., 2014).

However, there is reason to be optimistic about the potential in training particular mental skills for enhancing performance in areas of cognitive function that are dependent on those skills. Working memory (WM) training is an especially promising pathway given that a large body of evidence suggests that it is predictive of language comprehension and memory (Daneman and Merikle, 1996), reasoning (Kyllonen and Cristal, 1990; Süß et al., 2002), and fluid ability (Engle et al., 1999). While WM training has shown little evidence of transfer to improved intelligence (Shipstead et al., 2012; Melby-Lervaøag and Hulme, 2013; Melby-Lervaøag et al., 2016; Simons et al., 2016; Guye and Von Bastian, 2017), or at best, what amounts to 3 or 4 IQ points (Au et al., 2015), there is some evidence for near transfer, especially for complex span training, in which participants simultaneously engage in some ongoing processing task (e.g., sentence verification, arithmetic) and encode an element for later memory (Chein and Morrison, 2010), especially among older adults (Richmond et al., 2011; Carretti et al., 2013). Relationships between WM and language comprehension are robust (Daneman and Carpenter, 1983; Daneman and Merikle, 1996; Carretti et al., 2009), and there are strong theoretical reasons to believe that WM is a critical bottleneck for cognition that is exercised with language processing (Baddeley, 2003, 2012). In fact, in an experiment with older adults contrasting the effects of complex span training with an active verbal processing control, we demonstrated near transfer to unpracticed span tasks and far transfer to episodic memory for sentences, comprehension of sentences with temporary syntactic ambiguities, and verbal fluency (Payne and Stine-Morrow, 2017).

In contrast to training, engagement involves the implicit exercise of cognitive skills in the course of everyday activities (Stine-Morrow et al., 2014, 2021; Stine-Morrow and Manavbasi, 2022). Prospective longitudinal studies and natural experiments have provided some support for the idea that activity engagement, especially that involving cognitive and social stimulation, can reduce age-related cognitive declines as well as the risk of dementia (e.g., Wilson et al., 2002; Rohwedder and Willis, 2010; Mosca and Wright, 2018; Ihle et al., 2019). Experimental approaches in which participants are randomly assigned to complex environments (e.g., creative problem solving competition, challenging hobbies, community engagement) have provided clear demonstrations that psychometrically measured cognitive abilities can show growth through implicit practice in context (Carlson et al., 2008; Stine-Morrow et al., 2008, 2014; Park et al., 2014).

The activity engagement with the most robust consequences for cognitive resilience may be early-life education (Albert et al., 1995; Lövdén et al., 2020). Even with the challenges of disentangling correlations among education, socioeconomic status (SES), and selectivity, data from large-scale studies in cognitive epidemiology and prospective studies strongly suggest that education contributes directly to shaping intelligence in adulthood (Deary and Johnson, 2010, 2011; Ritchie et al., 2013) and a compression of declines in late life (Bennett et al., 2003; Le Carret et al., 2003; Scarmeas and Stern, 2003; Stern, 2009). A sizable percentage of autopsied brains of well-educated individuals who show no show clinical manifestation of the disease prior to death show evidence of Alzheimer’s-related pathology. There is also evidence that education buffers age-related decline in hippocampal volume (Noble et al., 2012). The mechanisms responsible for the “long arm” of education are unclear, but there is some evidence that effects may be mediated by ongoing activity engagement (Liu and Lachman, 2020), including literacy habits (Parisi et al., 2012).

Reading has long been recognized as a highly active form of mental engagement (Thorndike, 1917; Nell, 1988), but literacy practices have been largely neglected in the quest for cognitive enrichment to promote late-life cognitive health. There are good reasons to fill this gap. Readers allocate effort at the sentence level to process meaning, and at the discourse level to track the larger structures of argumentation and narratives and to mentally simulate events (Stine and Hindman, 1994; Stine-Morrow et al., 1996, 2001a,2001b,^2008^; Radvansky and Dijkstra, 2007; Gerrig and Jacovina, 2009; Noh and Stine-Morrow, 2009; Stine-Morrow and McCall, 2022). At the neural level, language processing depends on a language-specific core network, as well as domain-general networks that support memory, reasoning, executive control, mental simulation of sensorimotor experiences, and socioemotional processing (Ferstl et al., 2005; Speer et al., 2009; Barbey et al., 2014; Chow et al., 2014; Fedorenko and Thompson-Schill, 2014). While many aspect of discourse processing are maintained into late life (Stine-Morrow and Radvansky, 2017), longitudinal declines in discourse memory have been shown to track closely with declines in fluid ability (Payne et al., 2014b).

The systematic investigation of whether elective engagement in reading has a long-term impact on language processing and intellectual functions among literate individuals has been largely restricted to the study of children and college students. Reading experience is typically measured by checklists that assess recognition of unusual words, or the names of authors, magazines, or periodicals (Mol and Bus, 2011)—objective measures that are not subject to the social desirability effects of self-reports, and correlate with other measures of print exposure (e.g., number of books in the home, the ability to name a favorite author). Such measures have been found to be related to language processing abilities (e.g., speed of decoding, verbal fluency, comprehension) even when fluid ability is controlled (Stanovich and Cunningham, 1992). Longitudinal research has revealed lagged correlations with print exposure predicting language abilities and vice versa. In addition, there is evidence that the relationship between print exposure and language abilities increases through childhood to early adulthood. Such findings have prompted some to argue for a causal spiral between print exposure that contributes to more fluent reading, on the one hand, and abilities that afford access to an ever wider range of texts, on the other (Mol and Bus, 2011). In spite of correlational work cited above that is suggestive of the benefits of literacy engagement among older adults, to our knowledge, there has been no systematic investigation of literacy engagement as a pathway to cognitive enrichment for older adults.

The small body of work on the effects of print exposure in middle and late adulthood suggests that habitual literacy may have broad effects. Based on data from the Health and Retirement Study, Bavishi et al. (2016) reported that adjusting for education, health, and other covariates, individuals who read books showed a 20% reduction in mortality risk over 12 years relative to non-readers and those who read magazines and newspapers, an effect that was mediated by cognition. Print exposure can explain the increase in crystallized ability through adulthood (Stanovich et al., 1995). Controlling for education level, print exposure has been related to executive control, verbal fluency, and memory (Barnes et al., 2004). Older readers with higher levels of vocabulary process words more efficiently and allocate more attention to semantic processing (Stine-Morrow et al., 2008) and print exposure is predictive of more efficient lexical processing and greater allocation to conceptual integration, even when vocabulary is controlled (Payne et al., 2012a). Older adults with higher levels of print exposure are also more attuned to the statistical properties of syntactic structure (Payne et al., 2014a). Literacy often trumps educational level as a predictor of cognition in later life (Manly et al., 1999, 2004; Kavé et al., 2012), and has been shown to buffer the clinical manifestations of Alzheimer’s Disease (AD; Wilson et al., 2000). Finally, the well-replicated relationship between WM and text memory has been shown to be moderated by print exposure such that at the highest levels of print exposure, text recall is minimally constrained by poor WM (Payne et al., 2012a).

Only recently have the effects of reading on mind, brain, and well-being been explored using experimental approaches. Relatively short-term sessions of reading improve performance on both cognitive and affectively grounded theory-of-mind tasks (Kidd and Castano, 2013). An auditory narrative intervention with dementia patients produced improvement in auditory processing and memory relative to a passive retest control (Bartolucci and Batini, 2019).

A rapidly expanding literature in cognitive neuroscience is charting the effects of literacy on brain function (Fedorenko and Thompson-Schill, 2014). Changes in resting state functional connectivity among college students during and after reading a novel have been documented (Berns et al., 2013), suggesting that there may be relatively immediate effects of engaged reading on neural organization, which would be expected to prepare the mind to process subsequent experience. Recent findings suggest that literacy may strengthen connectivity between brain networks for language and executive cognitive; for example, engaging the visual word form area (VWFA) which serves as an interface between language processing and high-level vision (López-Barroso et al., 2020). This work demonstrates that functional connectivity between the VWFA and networks for executive control and vision are strongly tied to reading proficiency and age of acquisition, and therefore suggests that literacy engagement may promote the integration of cortical networks for language and executive control processes. Indeed, accumulating evidence suggests that the VWFA plays a central role in executive attention and WM resources necessary for word reading, providing evidence that literacy and WM rely upon shared neural mechanisms (Chen et al., 2019).

In addition to direct consequences for cognition and its neural substrates, habitual literacy may have the potential to engender habits of mental stimulation. Stine-Morrow and Manavbasi (2022) have argued that activity engagement can shape dispositions and attitudes that, in turn, shape the selection and experience of activities. In younger populations, reading engagement is related to the personality trait of openness (Medford and McGeown, 2012), a characteristic argued to support late-life cognitive health, in part, by expanding behavioral repertoires (Parisi et al., 2009; Hogan et al., 2012). There is evidence that experiential engagement can be shaped by activity engagement (Jackson et al., 2012; Stieger et al., 2020), and that the effects of activity engagement can be augmented among those whose existing temperaments are compatible with that activity (Payne et al., 2012b; Stine-Morrow et al., 2014; Cerino et al., 2020).

Collectively, this research suggests that reading, an accessible and cost-effective form of activity engagement, (a) can engender a highly active mental state so as to exercise a broad array of cognitive and neural processes, in particular, WM, episodic memory, and executive control, which are among the most vulnerable with aging, and (b) may be self-perpetuating in increasing the efficiency of lexical processing, enhancing effortful allocation to semantic processing, and shaping dispositions that sustain further literacy engagement. In this study, older adults with wide variation in initial cognitive status were randomly assigned to a literacy engagement group or to an active control. We examined whether a sustained period of reading engagement has measurable benefits for language processing and for cognitive skills underlying reading, as well as for dispositions that support cognitive health, in later adulthood.

## Materials and Methods

### Participants

In the interest of examining the effects of literacy engagement among older adults with a wide range in ability, we recruited from the community, assisted living residences, and a local memory clinic, and were conservative in screening out individuals experiencing early signs of mild cognitive impairment, Montreal Cognitive Assessment (MoCA; Nasreddine et al., 2005) > 16. Such natural variability in cognitive status allows for a more comprehensive view of cognitive aging (cf. Payne and Stine-Morrow, 2016). Other inclusion criteria for participation were: native English (or acquisition of English before age of 6 years); no severe sensory impairment that would limit participation; no stroke in the last 3 years; no current cancer treatment involving radiation or chemotherapy; no self-reported history of learning disability; relatively inactive (retired from paid employment for at least 6 months and < 20 h per week of scheduled activities); no engagement in a cognitive intervention in the last 2 years; and not already actively engaged in literacy activities and or games/puzzles (<10 h per week in both reading and gaming).

**FIGURE 1:**
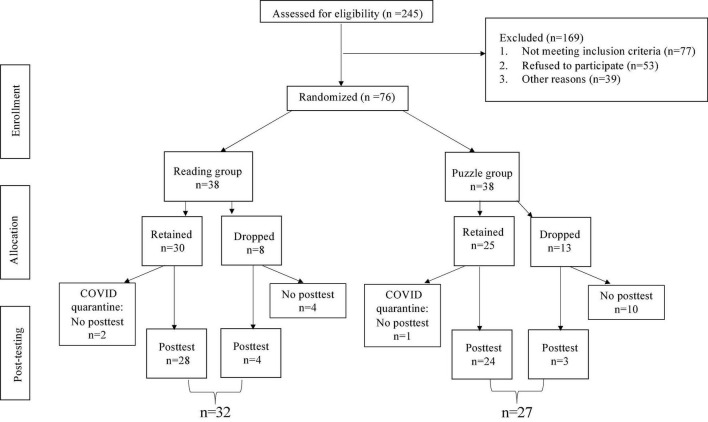
CONSORT diagram.

Based on an *a priori* power analysis using G*Power (Faul et al., 2007), we originally aimed for a sample size of 88. Assuming α = 0.05 (one-tailed) and reliability of within-subjects measures of ρ = 0.8, 44 participants per group would have afforded 0.90 power to detect a small effect size (Cohen’s *f* = 0.1). However, data collection was prematurely discontinued because of the COVID-19 pandemic. As shown in the CONSORT diagram in Figure 1, 76 adults (60–79 years of age; *M* = 68.7, *SD* = 5.7) agreed to randomization. Retention in the program for the Literacy group was 79%; and for the Control, 66%. Within these samples 3 people completed the program but could not return to the lab for post-test due to constraints of the pandemic. Seven individuals (4 from the Literacy group and 3 from the Control group) who dropped from the program, nevertheless, returned for post-test. Those who returned for post-test (*N* = 59) did not differ in age or education level from those who did not (*N* = 17), *p* > 0.1 for both; those who returned for post-test had higher scores at baseline on the MoCA (*M_*R*_* = 25.83, *SD* = 3.10; *M_*D*_* = 22.88, *SD* = 4.41), *t*(73) = 3.07, *p* = 0.003, *d* = 0.87, and on reading fluency (*M_*R*_* = 22.54, *SD* = 17.80; *M_*D*_* = 17.80, *SD* = 7.89), *t*(72) = 2.17, *p* = 0.033, *d* = 0.63, relative to those who did not. Any numerical differences in other cognitive measures (which generally favored those who returned) did not reach significance. There were no differences in personality traits at baseline between those who returned for post-test compared to those who dropped out. Table 1 shows performance at baseline for the sample that was retained to post-test. There was a trend for the Control group to report more time with reading engagement than the Literacy group at baseline, but otherwise the groups did not differ.

**TABLE 1:** Characterization of retained sample at baseline.

	Literacy engagement		Active control				
	(*n* = 32)		(*n* = 27)				
	*M*	*SD*	*M*	*SD*	*t*	*df*	*p*
Age	68.4	5.1	68.6	6.8	<1	57	
MoCA	26.2	3.3	25.4	2.8	<1	57	
Education level	15.5	2.3	15.1	2.6	<1	57	
Hrs reading/week	4.8	3.0	6.3	2.4	2.0	57	0.05
Hrs puzzles/week	3.0	3.1	3.6	4.3	<1	57	
WJ reading fluency	23.5	8.8	21.4	8.8	1.1	57	
**Cognition**							
Verbal ability	0.10	0.90	0.01	0.81	<1	57	
Print exposure	0.10	0.98	0.08	0.73	<1	57	
Working memory	0.09	0.87	0.04	0.85	<1	57	
Episodic memory	0.07	0.96	0.13	0.75	<1	57	
Verbal fluency	0.19	0.81	−0.01	0.77	<1	57	
**Personality**							
BF openness	3.74	0.81	3.76	0.67	<1	56	
BF conscientiousness	4.02	0.78	3.90	0.67	<1	56	
BF extraversion	3.23	0.77	3.19	0.68	<1	56	
BF agreeableness	4.32	0.39	4.33	0.33	<1	56	
BF neuroticism	2.23	0.82	2.34	0.68	<1	56	
Goldberg openness	3.62	0.69	3.60	0.57	<1	56	

### Measures

#### Cognition

*Verbal ability* was measured with the Advanced Vocabulary task (Ekstrom et al., 1976) and the North American Reading Test (NAART; Uttl, 2002), a = 0.91.

*Reading Fluency* was measured with an adapted version the Woodcock-Johnson IV Reading Fluency Test (Schrank et al., 2014), in which participants verify a series of simple sentences within 1 min.

*Print Exposure* was measured with the author Recognition Test (ART; Acheson et al., 2008), Magazine Recognition Test (MRT), and specialty versions of the ART focused on Fiction and Non-fiction (Mar and Rain, 2015), a = 0.90.

*Working Memory* was measured with three verbal span tasks (Conway et al., 2005), the category span, operation span, and reading span, a = 0.76. The *category span task* required participants to indicate whether or not a word belonged to a given category with a button press. After each category-word pair, participants were shown a letter and asked to recall it for a later memory test. Participants were given 5 s to respond. The *operation span task* required participants to decide whether a set of math problems was correct or incorrect. After each math problem, participants were shown a letter and asked to recall them at the end of the set. Participants were given 7 s to respond. The *reading span task* (Stine and Hindman, 1994) required participants to determine whether or not a sentence made sense. While making the judgments, they were also asked to remember the last word of the sentence. Eight seconds were given to complete this task. For each of these tasks, participants were asked to give equal weight to the decision task and the memory task. The number of items in a set increased across trials, with two sets per level. The task was discontinued when participants got two sets incorrect at a level. The score was given as the highest level completed correctly plus a fractional value representing the number correct at the next level.

*Episodic Memory* was assessed with the Hopkins Verbal Learning Test (HVLT; Hester et al., 2004). A composite measure was based on total recall across three trials and delayed recall after 20 min, a = 0.79.

*Verbal Fluency* (Tombaugh et al., 1999; Brickman et al., 2005) was assessed with a semantic fluency task in which participants provided as many exemplars as possible to each of three categories within 1 min, and a phonemic fluency task, in which participants generated as many words as possible beginning with each of three letters within 1 min, a = 0.73.

#### Personality

The MIDI Big Five Inventory (Lachman and Weaver, 1997) was included to measure of Openness to Experience, Conscientiousness, Extraversion, Agreeableness, and Neuroticism. Given our specific interest in the trait of Openness, we also administered Goldberg’s multifaceted inventory (Goldberg, 1999). This measure includes subscales to assess different aspects of Openness, including Intellect (e.g., has a rich vocabulary, enjoys thinking about things), Ingenuity (e.g., full of ideas), Competence (e.g., looks at the facts, meets challenges), Quickness (e.g., quick to understand things, enjoys reading challenging material), and Creativity (e.g., likes to solve complex problems, asks questions no one else does).

#### Sentence Processing and Memory

Participants read a series of 25 two-sentence passages word-by-word using the moving window method, and immediately recalled the gist of the passage aloud into a microphone. The sets of passages at pre-test and post-test dealt with topics in nature, geography, and history, and were comparable in length and complexity (Stine-Morrow et al., 2001a,^2008^). Reading times were analyzed for the first sentence only, with the second sentence serving as a buffer so that reading times for the sentence-final words were not contaminated by preparations for recall. Individual reading times were decomposed into process-related components using regression analysis (Miller and Stine-Morrow, 1998; Stine-Morrow et al., 2001a,^2008^, 2010a; Chin et al., 2015; Payne and Stine-Morrow, 2016; Ng et al., 2020). Briefly, word-level reading times for each individual were regressed onto text features reflecting processing demands (e.g., longer words take longer to read to accommodate decoding). Accordingly, the array of regression coefficients is taken to represent resource allocation (RA) to reading-related processes. Such coefficients for sentence-level processing are reliable at least across a 1-month interval and across different genres of text (Stine-Morrow et al., 2001a,^2008^). We were interested in the effects of literacy engagement in facilitating lexical processes and enhancing semantic integration processes (Stine-Morrow and McCall, 2022). Allocation to lexical processes was measured as time per syllable (decoding) and facilitation per log unit word frequency (lexical access). Clause- and sentence-final reading times are often longer than sentence-medial times; because RTs at these points increase with conceptual load and integration demands of the prior text (Aaronson and Scarborough, 1977; Haberlandt et al., 1986; Haberlandt and Graesser, 1989; Miller and Stine-Morrow, 1998; Stine-Morrow et al., 2010b), these times are taken to reflect meaning resolution at the end of syntactic constituents (Stine-Morrow and Payne, 2016). Data were excluded from one participant who was a stark outlier in allocating an average of 15 s to sentence-final words and otherwise read very quickly. Controlling for sentence-initial words, line breaks, and the introduction of new concepts, RA coefficients at baseline for these four components are reported in Table 2 (note that the RA coefficients for lexical access were multiplied by −1 to reflect the greater processing demands for infrequent words). There was an unexpected advantage in the control group for more time allocation to sentence wrap-up, but there was no difference between the groups in subsequent memory.

**TABLE 2:** Baseline values for resource allocation in reading.

	Literacy engagement		Active control				
	(*n* = 31)		(*n* = 27)				
	*M*	*SD*	*M*	*SD*	*t*	*df*	*p*
Decoding	105	109	148	106	1.7	55	
Lexical access	36	58	53	38	<1	55	
Intrasentence wrap-up	96	184	252	287	<1	55	
Sentence-final wrap-up	277	511	985	1515	2.3	55	0.03
Recall (%)	52	18	46	17	1.5	54	

Propositional recall was scored for gist criterion. Recall of the first sentence and the filler were highly correlated (>0.9) so we report recall for the first sentence alone (i.e., the text on which the RA analysis was based). Scoring reliability between pairs of scorers exceeded 0.85.

### Procedure

Participants were randomly assigned to 8 weeks of literacy engagement (i.e., novels, selected history, and biography) or to an active puzzle control. The program was entirely delivered *via* iPads on loan to participants from our lab. Options for the literacy engagement group were selected in collaboration with the Champaign Public Library Adult Literacy Specialist, who also advised in expanding our library for adults experiencing cognitive impairment. We purchased the reading materials and loaded them into the iBook app for the Literacy group; a variety of verbal puzzle apps were loaded onto the iPads for those in the Active Control. We developed an app for the iPads with a user-friendly interface that provided (a) a timer for participants to track adherence, (b) feedback on adherence, and (c) a log book to record reactions to the activities [e.g., ratings of enjoyment, challenge, and Flow (Payne et al., 2011); and answers to short open-ended questions to promote active engagement]. All interactions from the app were recorded on the iPad and downloaded as data when the iPad was returned to the lab. Participants were asked to spend 90 min per day, 5 days a week, across 8 weeks in their assigned activity. We did not screen for previous iPad use, but created an interface and support system with which participants seemed comfortable. The apps loaded on the iPad were limited to those participants would need for the study. Participants were given the lab phone number and email so that they could contact us if they had any questions. Pre-test and post-test batteries were administered in the lab.

## Results

### Drops, Adherence, and Experience With the Activity

First, we consider whether there were differences between participants who dropped (*n* = 21) and those who completed (*n* = 55) the 8-week program (irrespective of adherence or whether they returned for post-test). At baseline, those who were retained had higher scores on the MoCA (*M_*R*_* = 25.8, *SD* = 3.1; *M_*D*_* = 23.4, *SD* = 4.4), *t*(73) = 2.23, *p* = 0.035, *d* = 0.69, working memory (*M_*R*_* = 0.12, SD = 0.86; *M_*D*_* = –0.34, SD = 0.85), *t*(74) = 2.20, *p* = 0.031, *d* = 0.56, verbal fluency (*M_*R*_* = 0.17, *SD* = 0.82; *M_*D*_* = –0.49, *SD* = 0.93), *t*(72) = 2.95, *p* = 0.004, *d* = 0.79, and agreeableness (*M_*R*_* = 4.36, *SD* = 0.42; *M_*D*_* = 3.98, *SD* = 0.42), *t*(73) = 3.72, *p* < 0.001, *d* = 1.03, and trended higher for print exposure (*M_*R*_* = 0.12, *SD* = 0.85; *M_*D*_* = –0.31, *SD* = 0.85), *t*(74) = 1.97, *p* = 0.053, *d* = 0.50, and openness (*M_*R*_* = 3.80, *SD* = 0.76; *M_*D*_* = 3.39, *SD* = 0.69), *t*(69) = 1.95, *p* = 0.055, *d* = 0.54. There were no appreciable differences in this pattern between the literacy engagement and control groups.

Those who stayed with the program also showed higher scores on the Flow State Scale completed after each day’s activity (*M_*R*_* = 4.16, *SD* = 0.52; *M_*D*_* = 3.66, *SD* = 0.42), *t*(48) = 2.56, *p* = 0.014, *d* = 0.99, suggesting that the experience of deep immersion and a balance between skill and challenge may have supported maintenance of the activity. Ratings for enjoyment, interest, and challenge also numerically favored those who were retained, but none of these differences reached significance. There were too few dropped participants to test differences across groups, but the same numerical differences were apparent in each.

Adherence data during the program were available for 31 participants in the Literacy Engagement (LE) group and 25 in the Active Control (AC) group. If a participant were completely adherent, s/he would have engaged for a total of (1.5 × 5 × 8=) 60 h in the assigned activity. This criterion was met by 16 participants in the reading group and 15 participants in the control group. The mean number of hours spent in the assigned activity across the 8 weeks did not differ between the groups (*M_*LE*_* = 54.3, *SD* = 29.4; *M_*AC*_* = 48.6, *SD* = 32.1), *t* < 1. Groups also did not differ in self-reported enjoyment (*M_*LE*_* = 4.24, *SD* = 0.76; *M_*AC*_* = 4.26, *SD* = 0.49), *t* < 1, interest (*M_*LE*_* = 4.27, *SD* = 0.63; *M_*AC*_* = 4.15, *SD* = 0.50), *t* < 1, or Flow (*M_*LE*_* = 4.20, *SD* = 0.56; *M_*AC*_* = 3.95, *SD* = 0.49), *t* < 1. Those in the active puzzle control reported a greater sense of challenge relative to the reading group (*M_*LE*_* = 2.72, *SD* = 1.15; *M_*AC*_* = 3.89, *SD* = 0.66), *t*(48) = 4.38, *p* < 0.001, *d* = 1.20, however variability among participants in perceived challenge was greater in the reading group, *F* = 9.01, *p* = 0.004.

### Effects of Literacy Engagement on Cognition

**FIGURE 2:**
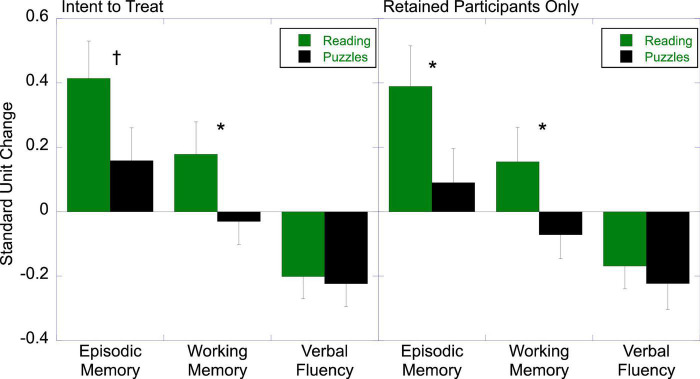
Effects of literacy engagement relative to the active control on cognition (^†^*p* < 0.06; *p < 0.05).

The left panel of Figure 2 shows the standard unit change in each of the cognitive domains, based on an intent-to-treat (ITT) analysis, which includes all participants for whom post-test data were available, regardless of adherence or retention in the program itself. A 2 (Treatment) by 3 (Ability) ANOVA on standard unit change revealed a main effect of Treatment, *F*_(1,57)_ = 4.30, *p* = 0.043, partial η^2^ = 0.070, showing more positive change in the Literacy Engagement group relative to the control. Even though the interaction was not significant, *F* < 1, we examined group differences in each ability given the difference among abilities, *F*_(2,114)_ = 15.30, *p* < 0.001, partial η^2^ = 0.212, and our *a priori* predictions. The Literacy group showed differential gains in working memory, *t*(57) = 1.69, *p* = 0.048 (one-tailed), *d* = 0.43, and a trend for gains in episodic memory, *t*(57) = 1.62, *p* = 0.055 (one-tailed), *d* = 0.42. Verbal fluency showed a decrease from pre-test to post-test that did not differ across groups, *t* < 1.

The ITT analysis represents a stringent test of hypothesis that takes into account participants’ ability and/or willingness to commit to activities for the duration of the program. We also analyzed the data to examine the effects of the treatment on the treated, that is, for the 28 individuals in the reading group and 24 individuals in the active control who nominally completed the program (regardless of levels of adherence). As in the whole sample, the groups did not differ in age, education level, or the cognitive abilities measured. These groups also did not differ in the total number of hours of program engagement (*M_*LE*_* = 60.5, *SD* = 25.9; *M_*AC*_* = 54.5, *SD* = 29.5), *t* < 1. As shown in the right panel of Figure 2, the Literacy Engagement group showed more positive change overall, *F*_(1,50)_ = 5.69, *p* = 0.021, partial η^2^ = 0.10. The effects on working memory, *t*(57) = 1.74, *p* = 0.045 (one-tailed), *d* = 0.47, and in episodic memory, *t*(57) = 1.78, *p* = 0.040 (one-tailed), *d* = 0.50, were a bit stronger, eking over conventional levels of significance. Total hours of engagement was not predictive of change in any of the three abilities in either group, *p* > 0.2.

### Effects of Literacy Engagement on Sentence Processing

**FIGURE 3:**
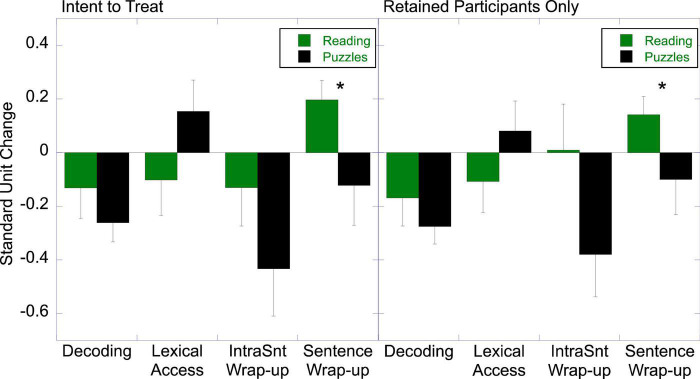
Effects of literacy engagement relative to the active control on resource allocation in sentence comprehension (*p < 0.05).

Our expectation was that sustained reading engagement would enhance efficiency of lexical processes (i.e., decrease RA to decoding and more facilitated processing for frequent words) and augment allocation to conceptual integration processes. Figure 3 shows the standard unit change in the four RA coefficients of interest. A 2 (Treatment) × 2 (Lexical Process) repeated measures ANOVA showed no difference between the groups, *F* < 1, in either the ITT analysis or for the retained sample. Participants generally showed facilitated decoding from pre-test to post-test, *t*(56) = 3.38, *p* < 0.001, *d* = 0.45, for the ITT analysis, and *t*(49) = 2.71, *p* = 0.009, *d* = 0.50, for the retained sample, perhaps reflecting a materials effects or a practice effect with the reading task, but the groups did not differ. There was also no difference between the groups in change in lexical access, *t*(55) = 1.52 for ITT, and *t*(55) = 1.43, for the ITT analysis and retained sample, respectively; in neither group did the change differ from zero, *t* < 1.33.

On the other hand, based on a 2 (Treatment) × 2 (Integration Process) repeated measures ANOVA, there was evidence for differential improvement in conceptual integration in the Literacy Engagement group relative to the Control, *F*_(1,55)_ = 5.15, *p* = 0.027, η^2^ = 0.086, in the ITT analysis, and *F*_(1,48)_ = 4.63, *p* = 0.036, η^2^ = 0.088, for the retained sample. The Literacy Engagement group showed increased sentence wrap-up at post-test relative to pre-test (i.e., change was greater than zero), *t*(30) = 2.05, *p* = 0.049, *d* = 0.37 for the ITT analysis, and *t*(27) = 2.73, *p* = 0.011, *d* = 0.51 for the retained sample, while the control group did not differ from baseline in either analysis, *t* < 1. The difference between the groups was significant regardless of whether the analysis was ITT, *t*(55) = 1.70, *p* = 0.048 (one-tailed), *d* = 0.45, or based on the retained sample, *t*(48) = 2.03, *p* = 0.024 (one-tailed), *d* = 0.58. Surprisingly, the control group showed a significant decrease in intrasentence wrap-up from pre-test to post-test, *t*(25) = 2.39, *p* = 0.025, *d* = 0.81, in the ITT analysis, and *t*(22) = 2.46, *p* = 0.022, *d* = 0.84, for the retained sample. The literacy engagement group showed no change, *t* < 1 for both the ITT analysis and retained sample. Nevertheless, the difference between the groups was not significant, in the ITT, *t*(55) = 1.65, or retained, *t*(48) = 1.35, sample. Collectively, there was some evidence that sustained reading engagement engendered small increases sentence-level integration.

### Effects of Literacy Engagement on Openness to Experience

**FIGURE 4:**
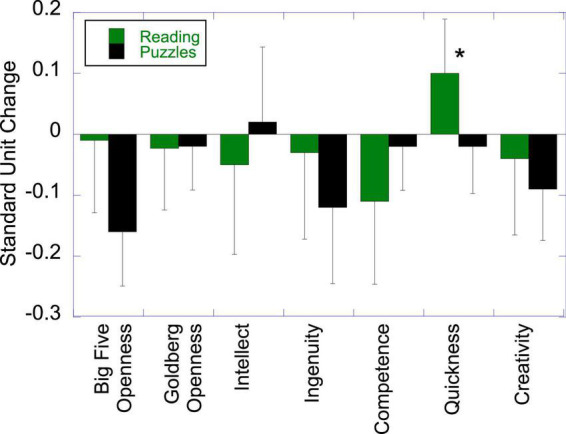
Effects of literacy engagement relative to the active control on openness to experience (*p < 0.05).

Figure 4 shows differences between the groups in change in Openness, based on the ITT analysis. Neither the BF measure nor the overall Goldberg measure showed differences between the groups *t* < 1 for both. The one isolated effect was on the Quickness facet of Goldberg scale, *t*(50) = 1.71, *p* = 0.047 (one-tailed), *d* = 0.30.

### Predictors of Adherence

We examined predictors of adherence among those who completed the program (see Table 3). Overall, those who showed higher levels of cognitive status and cognitive scores at baseline spent more time with their assigned activities. This was especially true of those in the active control group who engaged with verbal puzzles. Contrary to our expectation that those higher in openness to experience would be more drawn to reading, openness was not predictive of adherence (in either condition). Interestingly, extraversion was a negative predictor of time allocated to reading engagement, suggesting that more introverted individuals may have found it more comfortable to settle into a reading routine. Finally agreeableness was a negative predictor of time allocated to reading. The reasons for this are unclear, but reading does require sustained attention over time, which may be difficult to maintain among those who are more accommodative to the needs of others.

**TABLE 3:** Predictors of adherence (correlations between baseline variables and total hours of activity).

	Overall	Literacy engagement	Active control
	(*n* = 49)	(*n* = 27)	(*n* = 22)
Age	0.22	**0.39**	0.10
Ed level	0.15	0.26	0.00
MoCA	**0.50**	**0.45**	**0.59**
Verbal ability	0.22	0.28	0.14
Print exposure	0.20	0.21	0.17
Working memory	**0.40**	0.19	**0.64**
Episodic memory	**0.36**	0.23	**0.57**
Verbal fluency	**0.32**	0.31	0.32
BF openness	–0.16	–0.14	–0.18
BF conscientiousness	–0.18	–0.35	0.05
BF extraversion	–0.26	−**0.38**	–0.11
BF agreeableness	–0.31	−**0.42**	–0.17
BF neuroticism	–0.04	0.15	–0.30
Goldberg openness	–0.15	–0.17	–0.21

*Bolded values p < 0.05.*

### Predictors of Experimental Effects

**FIGURE 5:**
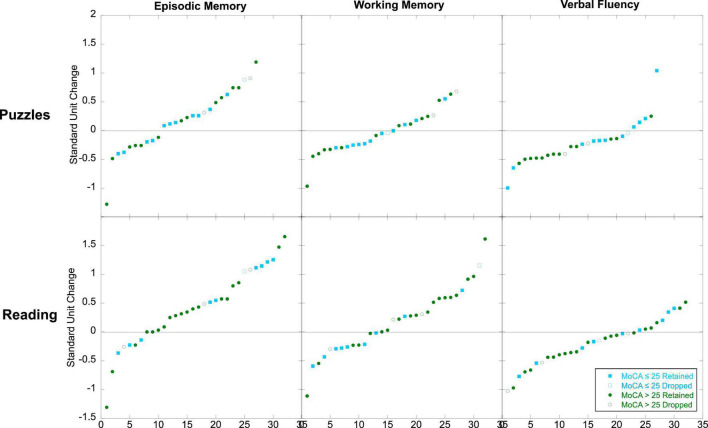
Individual differences in cognitive change for those with MoCA scores above 25 (green) and with MoCA scores at or below 25 (blue). The x-axis is the rank order of change.

We also examined the extent to which baseline characteristics were predictive of change in cognition among those who completed the program. As shown in Table 4, neither age nor cognitive status (MoCA) was related to changes in cognitive scores. Figure 5 plots the rank order of individual differences in change distinguishing those who were above and below the typical cutoff score (MoCA > 25 vs. ≤ 25) indicating possible cognitive impairment. This figure further illustrates that gains in episodic memory and WM were achievable by individuals who may typically be screened out from participating in cognitive enrichment research.

**TABLE 4:** Predictors of change [correlations between baseline characteristics and change (Δ) in cognition].

	ΔWorking memory		ΔEpisodic memory		ΔVerbal fluency	
	Literacy engagement	Active control	Literacy engagement	Active control	Literacy engagement	Active control
	(*n* = 32)	(*n* = 27)	(*n* = 32)	(*n* = 27)	(*n* = 32)	(*n* = 27)
Age	–0.14	0.19	0.16	0.08	–0.16	–0.32
Education level	0.08	0.18	–0.01	−**0.48**	0.04	–0.09
MOCA	0.12	0.02	–0.06	–0.08	–0.15	–0.26
Print exposure	0.05	–0.10	–0.09	–0.08	−**0.39**	–0.20
Verbal ability	0.00	0.00	–0.07	–0.04	–0.04	–0.32
BF openness	0.29	0.18	–0.14	–0.22	–0.11	0.27
BF conscientiousness	–0.04	0.14	–0.07	−**0.41**	–0.26	0.37
BF extraversion	0.31	0.21	–0.07	–0.21	–0.06	**0.58**
BF agreeableness	0.10	–0.10	–0.13	−**0.44**	–0.24	**0.45**
BF neuroticism	0.24	0.02	0.11	–0.06	0.17	−**0.39**
Goldberg openness	0.17	0.20	–0.15	–0.18	–0.09	0.00

*Bolded values p < 0.05.*

As shown in Table 4, one isolated effect in the literacy group was that there was less decline in verbal fluency among those with lower print exposure at baseline, suggesting greater benefits among those with less developed literacy habits at baseline. We also note that there were sporadic correlations between baseline scores and cognitive change within the control group (presumably reflecting moderators of simple re-test effects, for which we have no explanation).

## Discussion

There is long-term interest in the extent to which language engages language-specific vs. domain-general processes (Just and Carpenter, 1992; Caplan and Waters, 1999; Fedorenko and Thompson-Schill, 2014), and under what conditions (Wingfield and Grossman, 2006). We found evidence that sustained engagement with fiction over an 8-week period impacted reading processes, as well as WM and episodic memory, domain-general areas of cognition that are among the most vulnerable with aging. This offers promise for cognitive enrichment, but also suggests that reading engages these domain-general capacities.

### The Promise of Literacy and Language Engagement for Offsetting Cognitive Impairment

Within the Ecological Model of cognitive aging (Stine-Morrow et al., 2014, 2021; Stine-Morrow and Manavbasi, 2022), behavioral engagement—as the commitment to activities over time—affords opportunities for deeper forms of engagement. Activities vary in the specific cognitive processes engaged (“attentional engagement”), so that patterns of growth in skill and knowledge are assumed to be constrained by the constellation of processes implicitly exercised by the activity. In the short-term, attentional engagement in the course of ordinary activities (e.g., leisure reading) can have localized effects on cognition. Consistent with the view that activities can implicitly exercise cognitive processes specific to the activities engaged (Stine-Morrow et al., 2014), gains were isolated to memory processes, which are well-established as central to narrative comprehension (Daneman and Merikle, 1996; Noh and Stine-Morrow, 2009) and engage shared neural mechanisms with language processing (Chen et al., 2019; López-Barroso et al., 2020). Given theories of language processing suggesting that production processes are critical to comprehension (Federmeier, 2007), it might have been expected that verbal fluency would have also benefited. We found no evidence for this. The empirical evidence for production processes in comprehension primarily resides at the word level in studies of sentence understanding. It may be that production is less engaged at the discourse level. Our findings present an interesting contrast to those from a study by Cerino et al. (2020), who found a 6-week conversation intervention to improve several measures of executive control, including verbal fluency. While leisure reading may not specifically exercise production processes, there are other contexts for language use that certainly do.

While the immediate effects of engagement may be specific to the cognitive components exercised, incremental gains in efficiency and integration in cognitive modules allow for expanded access to new areas of the cognitive ecology (e.g., new authors, expanded literacy forms, novel content and perspectives in the context of social engagement). Furthermore, selective growth in cognitive skills may potentiate plasticity for related skills (”mutualism”; van der Maas et al., 2006, 2017). Through this ongoing process of cognitive growth and ecological affordances, there is potential for cognitive enhancement on a broader scale. Relative to other lifestyle activities, the cognitive processes underlying reading are somewhat well-understood, thus making it a potentially useful probe for studying plasticity through an ecological lens. As an activity with potential to build both crystallized abilities (Stanovich et al., 1995) and as suggested in our data, selected fluid abilities, leisure reading may offer considerable promise for cognitive enrichment.

### The Plasticity of Reading Processes

The nature of language comprehension is that it allows for the creation of mental representations that establish associations among previously unrelated concepts (Ratcliff and McKoon, 1978). Outside a language context, an associative deficit is well-replicated among older adults (Naveh-Benjamin, 2000). Within language, older adults may take longer to encode associations, but once encoded, retrieval of these associations appears to be quite robust (Howard et al., 1986; Stine and Hindman, 1994). Unlike lexical access, which is automatic and obligatory (Fodor, 1983), there is considerable variability in the extent to which readers engage integration processes (Stine-Morrow and Payne, 2016; Stine-Morrow and McCall, 2022). Allocation to conceptual integration appears to be enhanced among those with high levels of verbal ability (Stine-Morrow et al., 2008; Chin et al., 2015); proficiency in skilled reading (Ng et al., 2020); print exposure (Payne et al., 2012a); and domain-related knowledge, which presumably increases integration demands (Miller et al., 2004; Chin et al., 2015). The current study adds to this mostly correlational literature in providing experimental support for the idea that reading is a skill that continues to develop as a function of reading engagement (Mol and Bus, 2011). Building on earlier research showing that conceptual integration can be enhanced through instruction within a relatively short timeframe (cf. Stine-Morrow et al., 2010a), the current study suggests that integrative processing may be improved through sustained leisure reading of fiction. The precise mechanisms through which leisure reading engenders more sentence-level integration is not clear, but assuming that integration supports the ongoing consolidation of the text representation (Stine-Morrow et al., 2001a,b, 2006, 2008; Ng et al., 2020), one possibility is that the narrative experience is more satisfying when effort is allocated to conceptual integration, thereby shaping the allocation policy over time.

By contrast, we found no evidence for effects on word-level processes. High-verbal readers (Stine-Morrow et al., 2008) and those with higher levels of reading proficiency (Landi, 2010; Ng et al., 2020; Payne et al., 2020) and print exposure (Payne et al., 2012a) do show facilitated lexical processing. However, this advantage likely arises from extensive exposure to lexical items in diverse contexts. For example, among proficient readers, word reading times are sensitive to orthographic and phonological neighborhoods (Payne et al., 2020), suggesting that skilled reading is supported by a complex lexical network of semantic and surface features. As such, 8 weeks of reading may be insufficient to measurably enhance lexical recognition processes.

### Plasticity in Personality

There is some evidence that openness can be enhanced by intellectual engagement (Jackson et al., 2012). More generally, contrary to the conceptualization of personality as “set in plaster” (James, 1890/1950), there is growing recognition that traits can be shaped gently over time through experience (Roberts et al., 2003). Nevertheless, the effects of experience on openness do appear to be small and somewhat fragile (Roberts et al., 2017). We found an isolated effect of differential growth from baseline (a little more than a tenth of a standard deviation) in the Quickness facet of Openness, but certainly no robust effect at the trait level.

### Who Benefits?

Those with lower levels of cognitive performance at baseline were less likely to stick with the program, and if they were retained, they were less likely to adhere. However, this was more pronounced in the puzzle group than in the literacy engagement group. One implication is that literacy may be enjoyable to individuals with a wider range of cognitive skills relative to puzzles. Contrary to our expectation, we found no evidence that those with higher levels of Openness were more likely to sustain reading engagement or that the effects of literacy engagement were moderated by Openness. Nevertheless, questions about how dispositional fit to behavioral engagement are worth pursuing (Stine-Morrow et al., 2014; Cerino et al., 2020; Stine-Morrow and Manavbasi, 2022). Finally, we note that those who experienced more Flow during the program activities were more likely to be retained, providing evidence for the assumption that sustaining engagement will depend on how the activity is experienced (Hess, 2014; Worm and Stine-Morrow, 2021).

### Strengths and Limitations

An important strength of study is the design, which employed an active control that equated interaction with lab personnel, use of electronic media, and the availability of choice, in addition to practice effects on the criterion measures. We did not employ a passive control group. There is some controversy about what makes for an effective control group in behavioral interventions (Freedland et al., 2019). Consistent with principles articulated by Freedland et al., our control group was selected to isolate mechanistic effects—specifically of literacy engagement. Both groups showed some improvement in episodic memory, and without a passive control it is impossible to know whether improvement in the control group was attributable to practice effects or interactions with a (perhaps novel) technology. Given earlier work showing the effect of iPad use on episodic memory (Chan et al., 2016), it is quite plausible that new memory demands associated with navigating the iPad interface contributed to memory improvement. Importantly, the active control group enabled us to establish beneficial effects of literacy engagement above and beyond those associated with media use.

Another strength is that outcome measures included both assessments of language processing as well as of domain-general abilities thought to underpin language comprehension. Thus, we were able to establish some benefit from the activity directly engaged as well broader cognitive abilities engaged by those processes (i.e., “near” and “far” transfer).

Finally, the sample included older adults with relatively poor cognitive status (those who are typically screened out from cognitive aging research). While such individuals were more likely to prematurely withdraw from the study, those who were retained showed similar benefits, suggesting that leisure reading may support memory skills even among adults who are already starting to experience significant declines.

There are limitations. More extensive measurement of both cognition, especially executive control, and language processes (e.g., using eye-tracking and electrophysiological methods) are needed to provide a more complete and more nuanced understanding of how everyday reading shapes mind and brain. Also, due to time constraints, we did not include a measure of discourse processing. Perhaps most significantly, data collection was cut short by the COVID-19 pandemic. The consequent sample was smaller than we had planned and did not allow for a particularly robust test of the hypothesis for enrichment effects of literacy on cognition, and especially limited our ability to detect individual differences in effects. We did not correct for multiple comparisons across domains of function, and (because of the small sample) effects do not survive such a correction.

## Conclusion

In spite of these limitations, our study offers provocative pilot data that provides encouragement for future research. Education early in the lifespan, built on literacy within a structured social context, is among the most powerful contributors to cognition throughout the lifespan. While available in some form, educational experiences are often more difficult to arrange in later adulthood (Riley and Riley, 2000). The cultivation of literacy habits can occur at any age, and literacy engagement may be more easily incorporated into daily routines than formal education. Many questions remain to be addressed, such as the relative advantages of engagement with different genres of text [e.g., fiction and non-fiction (Mar et al., 2006; Mar, 2011)]; the effects of reading on the development of knowledge and reciprocal effects between knowledge growth and comprehension processes (Troyer and Kutas, 2020; Troyer et al., 2020), and the ways in which literacy engagement and its effects may be shaped by social context. Parallel questions regarding the neurobiology of language comprehension also persist, motivating further examination of the neural mechanisms underlying social, cognitive, and affective processes engaged through literacy (Friedman and Miyake, 2000; Speer et al., 2009; Chow et al., 2014; Fedorenko and Thompson-Schill, 2014). An understanding of how sustained reading impacts cognition (and its neural underpinnings) will not only contribute to the science of enrichment effects with aging, but also to the science of language comprehension.

## Data Availability Statement

The datasets presented in this study can be found in online repositories. The names of the repository/repositories and accession number(s) can be found below: https://osf.io/e4mxv/.

## Ethics Statement

The studies involving human participants were reviewed and approved by Office for the Protection of Human Subjects University of Illinois. The patients/participants provided their written informed consent to participate in this study.

## Author Contributions

ES-M, AB, and DL conceptualized the study and collaborated in study design and interpretation. SN organized the measurement protocol and recruitment. SN, GM, and IM tested participants, scored protocols, and assisted with data management. ES-M and GM analyzed data. ES-M wrote the manuscript. All authors reviewed and/or edited before submission.

## Conflict of Interest

The authors declare that the research was conducted in the absence of any commercial or financial relationships that could be construed as a potential conflict of interest.

## Publisher’s Note

All claims expressed in this article are solely those of the authors and do not necessarily represent those of their affiliated organizations, or those of the publisher, the editors and the reviewers. Any product that may be evaluated in this article, or claim that may be made by its manufacturer, is not guaranteed or endorsed by the publisher.
